# Efficacy and Safety of Neoadjuvant Monoimmunotherapy With PD-1 Inhibitor for dMMR/MSI⁃H Locally Advanced Colorectal Cancer: A Single-Center Real-World Study

**DOI:** 10.3389/fimmu.2022.913483

**Published:** 2022-07-25

**Authors:** Xuan Zhang, Renfang Yang, Tao Wu, Xinyi Cai, Guoyu Li, Kun Yu, Yong Li, Rong Ding, Chao Dong, Jinsha Li, Ruixi Hu, Qing Feng, Yunfeng Li

**Affiliations:** ^1^ Department of Colorectal surgery, Yunnan Cancer Hospital, The Third Affiliated Hospital of Kunming Medical University, Kunming, China; ^2^ Department of Hepatobiliary and Pancreatic Surgery, Yunnan Cancer Hospital, The Third Affiliated Hospital of Kunming Medical University, Kunming, China; ^3^ Department of Minimally Invasive Intervention, Yunnan Cancer Hospital, The Third Affiliated Hospital of Kunming Medical University, Kunming, China; ^4^ Department of Oncology, Yunnan Cancer Hospital, The Third Affiliated Hospital of Kunming Medical University, Kunming, China

**Keywords:** locally advanced colorectal cancer, neoadjuvant immunotherapy, programmed cell death protein-1 inhibitor, mismatch repair-deficient, microsatellite instability-high (MSI-H)

## Abstract

**Objective:**

To explore the efficacy and safety of single-agent programmed cell death protein-1 (PD-1) inhibitor in the neoadjuvant treatment of patients with mismatch repair-deficient (dMMR) or microsatellite instability-high (MSI-H) locally advanced colorectal cancer (LACRC) through single-center large⁃sample analysis based on real⁃world data in China.

**Methods:**

This study was a retrospective, single-center, case series study. 33 colorectal cancer (CRC) patients with clinical stage of T3~4N0~2M0 treated in Yunnan Cancer Hospital from June 2019 to June 2021 were analyzed retrospectively. Among them, 32 patients were dMMR or MSI-H or both dMMR and MSI-H, and one patient was both dMMR and microsatellite stability (MSS) (excluded in the final analysis). All 32 patients received neoadjuvant immunotherapy (nIT) with single-agent PD⁃1 inhibitor.

**Results:**

Among the 32 patients, 8 (25%) were locally advanced rectal cancer (LARC) and 24 (75%) were locally advanced colon cancer (LACC); 4 (12.55%) were stage II and 28 (87.5%) were stage III. The median number of cycles of 32 patients with dMMR/MSI-H LACRC receiving nIT with single-agent PD-1 blockade was 6 (4~10), and the median number of cycles to achieve partial response (PR) was 3 (2~4). Among them, three LARC patients achieved clinical complete response (cCR) and adopted the watch-and-wait (W&W) strategy. The objective response rate (ORR) of the other 29 patients with radical surgery was 100% (29/29), the pathological response rate was 100% (29/29), the rate of major pathological response (MPR) was 86.2% (25/29), and the rate of pathological complete response (pCR) was 75.9% (22/29). The incidence of immune-related adverse events (irAEs) in 32 patients during nIT was 37.5% (12/32), while the incidence of irAEs in 22 patients with operation during adjuvant immunotherapy was 27.3% (6/22), all of which were grade 1~2. No grade 3 or above irAEs were occured. The median time from the last nIT to surgery was 27 (16~42) days. There were no delayed radical resection due to irAEs in these patients. All 29 patients achieved R0 resection. The incidence of surgical-related adverse events (srAEs) in perioperative period was 10.3% (3/29).

**Conclusions:**

Neoadjuvant monoimmunotherapy with PD-1 inhibitor has favorable ORR and pCR rate, and relatively low incidences of irAEs and srAEs for patients with dMMR/MSI-H LACRC, suggesting that this nIT regimen of single-agent PD-1 inhibitor is significantly effective and sufficiently safe.

## Introduction

The latest global cancer statistics displayed that colorectal cancer (CRC) is one of the most frequent malignancies, and that CRC ranked third in incidence rate and second in mortality rate (1). The great majority of CRC patients are in the stage of local progression upon diagnosis, which makes effective treatment more hard. Locally advanced colorectal cancer (LACRC) is defined as CRC stage II (clinical T3 to 4, N0) and stage III (any clinical T, N1 to 2). In recent years, advancements in standardized surgery and subsequent enhancements in neoadjuvant therapies have improved the survival and prognosis of LACRC patients. Nevertheless, surgical complications, and adverse events (AEs) caused by neoadjuvant chemotherapy (nCT) or neoadjuvant chemoradiotherapy (nCRT) remain significant and inevitable problems.

With the advent of the era of precision medicine, scientists have begun to explore the impact of microsatellite status on the tumor characteristics (2). Previous studies have found that mismatch repair-deficient (dMMR) or microsatellite instability-high (MSI-H) CRC accounts for 10% ~ 15% of all CRC, mostly colon cancer, and only 5% of rectal cancer (3). Among them, dMMR/MSI⁃H metastatic CRC (mCRC) accounted for only 4%, while the proportion of dMMR/MSI⁃H LACRC increased to 12% ~ 20% (4).

What is the efficacy of current neoadjuvant therapies for patients with MSI-H/dMMR LACRC? With regard to nCT for dMMR/MSI⁃H LACRC, the FOxTROT study indicated that there was no tumor regression and no benefit in 2-year survival after nCT for dMMR locally advanced colon cancer (LACC) (5). Meanwhile, a retrospective analysis from Memorial Sloan Kettering Cancer Center demonstrated that almost 30% of dMMR locally advanced rectal cancer (LARC) treated with nCT exhibited disease progression (6).These two studies thus suggested that dMMR/MSI-H LACRC has obvious resistance to nCT. In terms of nCRT for dMMR/MSI⁃H LARC, this study reported that the pathological complete response (pCR) rate of dMMR LARC after nCRT was 14% (6). Meanwhile, a retrospective study indicated that the tumor downstaging rate of dMMR LARC patients after nCRT was 70% (7). Besides, another study demonstrated that the pCR rate of dMMR LARC after nCRT was up to 27.6% (8). The above studies illustrated that dMMR/MSI-H LARC is sensitive to nCRT. Nevertheless, to some extent, nCRT can leads to post-surgical morbidities, such as anastomotic leakage and poor healing of the perineal wound, as well as long-term organ and function damage, such as urination and sexual dysfunction, as well as loss of anal sphincter function.

Fortunately, the emergence of neoadjuvant immunotherapy (nIT) brings new hope to these patients (9). The NICHE study published in *Nature Medicine* in 2020 has demonstrated that the pCR rate of 60% (12/20) in the treatment of dMMR/MSI-H stage I-III colon cancer with nivolumab combined with ipilimumab (10). This trial shows that immune checkpoint inhibitors (ICIs) may have great application space in the comprehensive treatment decision-making of non-mCRC in the future. Meanwhile, the PICC study published in *Lancet Gastroenterol Hepatol* in 2021 suggested that the pCR rate of initially resectable dMMR/MSI-H LACRC treated with treprizumab alone could reach 65% (11). This study has largely promoted the application of immunotherapy in the neoadjuvant setting for LACRC patients with dMMR/MSI-H.

Notwithstanding, data on nIT for LACRC are still scarce. Therefore, our center conducted a retrospective exploratory analysis to evaluate the safety and effectiveness of nIT with single-agent programmed cell death protein-1 (PD-1) inhibitor for dMMR/MSI-H LACRC.

## Materials and Methods

### Patients Selection

This study is a retrospective, single center, case series study. The clinical data of 32 patients with clinical stage T3~4N0~2M0 dMMR/MSI-H CRC treated in Yunnan cancer hospital/The Third Affiliated Hospital of Kunming Medical University from June 2019 to June 2021 were collected excluding one patient with both dMMR and microsatellite stability (MSS). The baseline demographic and clinical characteristics of all patients with dMMR/MSI-H LACRC are indicated in **Table 1**. All data collected in this study have obtained informed consent. This study has received the full approval of the ethics committee of Yunnan cancer hospital/The Third Affiliated Hospital of Kunming Medical University.

**Table 1:** Baseline demographic and clinical characteristics of patients with dMMR/MSI-H LACRC.

	nIT with PD-1 inhibitor group
	(n=32)
Age, years	44(23-62)
Sex	
Female	15/32 (46.88%)
Male	17/32 (53.13%)
ECOG performance status	
0	18/32 (56.25%)
1	14/32 (43.75%)
Gene detection of LS	9
LS associated gene mutation detected	3/9
MSH2 mutation	2
MLH1 mutation	1
LS associated gene mutation not detected	6/9
Suspected LS without genetic testing	5
Personal history of endometrial cancer	2/5
Family history of CRC	2/5
Family history of extra-intestinal malignancies	1/5
Previously received nCT	6/32 (18.75%)
Previously received nCRT	2/32 (6.25%)
Primary tumor location	
Ascending colon	7/32 (21.88%)
Hepatic flexure	4/32 (12.55%)
Transverse colon	4/32 (12.55%)
Splenic flexure	2/32 (6.25%)
Descending colon	2/32 (6.25%)
Sigmoid colon	2/32 (6.25%)
Rectosigmoid junction	3/32 (9.38%)
Rectum	8/32 (25%)
Clinical T stage	
T3	6/32 (18.75%)
T4	26/32 (81.25%)
Clinical N stage	
N0	4/32 (12.55%)
N1	5/32 (15.63%)
N2	23/32 (71.88%)
Clinical TNM stage	
II	4/32 (12.55%)
III	28/32 (87.5%)
Histological appearance	
Well differentiated	9/32 (28.13%)
Moderately differentiated	13/32 (40.63%)
Poorly differentiated	10/32 (31.25%)
Loss of expression of MMR proteins	
MLH1 only	2/26 (7.69%)
MSH2 only	5/26 (19.23%)
MSH6 only	1/26 (3.85%)
PMS2 only	4/26 (15.38%)
MLH1 and PMS2	10/26 (38.46%)
MSH2 and MSH6	3/26 (11.54%)
MSH2, MSH6 and PMS2	1/26 (3.65%)
Not tested	6 (18.75%)
MSI status	
MSI-H	17
Not tested	15
KRAS status	
Mutant type	6/17 (35.29%)
wild type	11/17 (64.71%)
NRAS status	
Mutant type	2/17 (11.76%)
wild type	15/17 (88.24%)
BRAF V600E status	
Mutant type	1/17 (5.88%)
wild type	16/17 (94.12%)

LACRC, Locally advanced colorectal cancer; LARC, Locally advanced rectal cancer; dMMR, Mismatch repair-deficient; MSI-H, Microsatellite instability-high; ECOG, Eastern cooperative oncology group; LS, Lynch syndrome; CRC, Colorectal cancer; nIT, neoadjuvant immunotherapy; nCT, neoadjuvant chemotherapy; nCRT, neoadjuvant chemoradiotherapy; TNM, Tumor Node Metastasis; MMR, mis-match repair; MSI, microsatellite instability.

One LARC patient who received nIT in this study was both dMMR and MSS, so the patient was not included in the baseline analysis.

### Inclusion Criteria

1) Age from 18 to 75 years old;
2) Diagnosed with CRC by pathology;
3) The clinical stage of initial diagnosis by imaging is T3~4N0~2M0;
4) Confirmed as dMMR by immunohistochemistry (IHC) or MSI-H by polymerase chain reaction (PCR);
5) Eastern Cooperative Oncology Group (ECOG) physical status score ≤ 1 (12);
6) Treatment with neoadjuvant anti-PD-1 monotherapy (no manufacturer limited), regardless of whether nCRT or nCT has been received before;
7) Have not received radical surgery for this CRC before;
8) No history of biological therapy, immunotherapy or other experimental drug therapy;
9) Not accompanied by systemic infection requiring antibiotic treatment;
10) Not combined with immune system diseases.

### Exclusion Criteria

1) One patient with discordant mis-match repair (MMR) and microsatellite instability (MSI) testing was excluded from the current analysis.

### Data Collection

The data includes complete basic information of patients, serum carcinoembryonic antigen (CEA), colonoscopy, pathological biopsy, chest/abdomen/pelvic enhanced CT, pelvic high-resolution MRI, MMR proteins expression, and MSI status, etc. Efficacy evaluation and subsequent treatment approaches including surgery or watch-and-wait (W&W) strategy, and postoperative pathological outcomes including pTNM staging and tumor regression grade (TRG) grade were also recorded.

### Treatment Methods

All 32 patients who met the inclusion and exclusion criteria received nIT with single-agent PD-1 inhibitor, of which 4 patients used pembrolizumab, 9 patients used sintilimab and 19 patients treated with tiselizumab. Within the first day of each treatment cycle, they received nIT with intravenous drip of 200mg PD-1 inhibitor. The duration of each cycle of treatment was 3 weeks, regardless of how many courses of treatment were used (200 mg IV Q3W). Before using nIT, regardless of whether you have received nCT or nCRT. All patients underwent radical resection or W&W strategy. Of the 29 patients who underwent surgery, 22 received adjuvant immunotherapy with PD-1 blockade.

### Observation Indicators and Evaluation Criteria

#### Evaluation Indicators of Imaging Efficacy After nIT

Objective response rate (ORR): The proportion of patients whose tumor volume shrinks to a predetermined value and can maintain the minimum time limit is the sum of the proportions of complete response (CR) and PR, that is, ORR=CR+PR. The efficacy of nIT were assessed by the Response Evaluation Criteria in Solid Tumors RECIST Version 1.1 (RECIST 1.1) (13).

#### Evaluation Indicators of Pathological Efficacy After nIT

##### Tumor Regression Grade

According to the TRG of National Comprehensive Cancer Network (NCCN) (14).

##### Pathological Complete Response

Defined as tumor regression induced by neoadjuvant therapy, there is no residual cancer cells or positive lymph nodes in pathology (pCR=TRG-0) (14).

##### Major Pathological Response

Considered as tumor regression induced by neoadjuvant therapy, with pathological residual tumor ≤ 10% (PCR = TRG-0 + TRG-1) (14).

#### Adverse Events During Immunotherapy

Refers to the AEs related to preoperative nIT and/or postoperative adjuvant immunotherapy that occurred from the beginning of nIT to the end of follow-up. Treatment-related adverse events were assessed by the Common Adverse Event Evaluation Criteria (CTCAE) version 5.0 published by the US Department of Health and Human Services (15). Among them, immune-related adverse events (irAEs) were evaluated according to literature standards (16).

#### Operation and Surgical-Related Adverse Events

The time from the end of nIT to surgery was defined as the time from the end of the last administration to surgery. Perioperative complications refer to complications directly or indirectly related to surgery that occurred from the day of surgery to 30 days after surgery. The grading of surgical complications was based on the Clavien-Dindo grading evaluation standard (17).

### Follow Up Methods

Both patients who underwent surgery and those who adopted the W&W strategy were followed up every 3 months within 2 years, and every 6 months thereafter. During each follow-up, routine blood test, CEA, and enhanced CT examination of chest, abdomen and pelvis. were required for all patients. On this basis, patients underwent W&W strategy need to add the pelvic MRI, transrectal ultrasound and digital rectal examination. If there are suspicious lesions, colonoscopy and biopsy should be added. The last follow-up time was March 31, 2022.

### Statistical Analysis

All data were processed using SPSS 24.0 statistical software. Measurement data were represented by M (range). Categorical variables were compared using the χ2 test or Fisher’s exact test. The difference was statistically significant with P<0.05.

## Results

### Characteristics of the Patients

**Figure 1:**
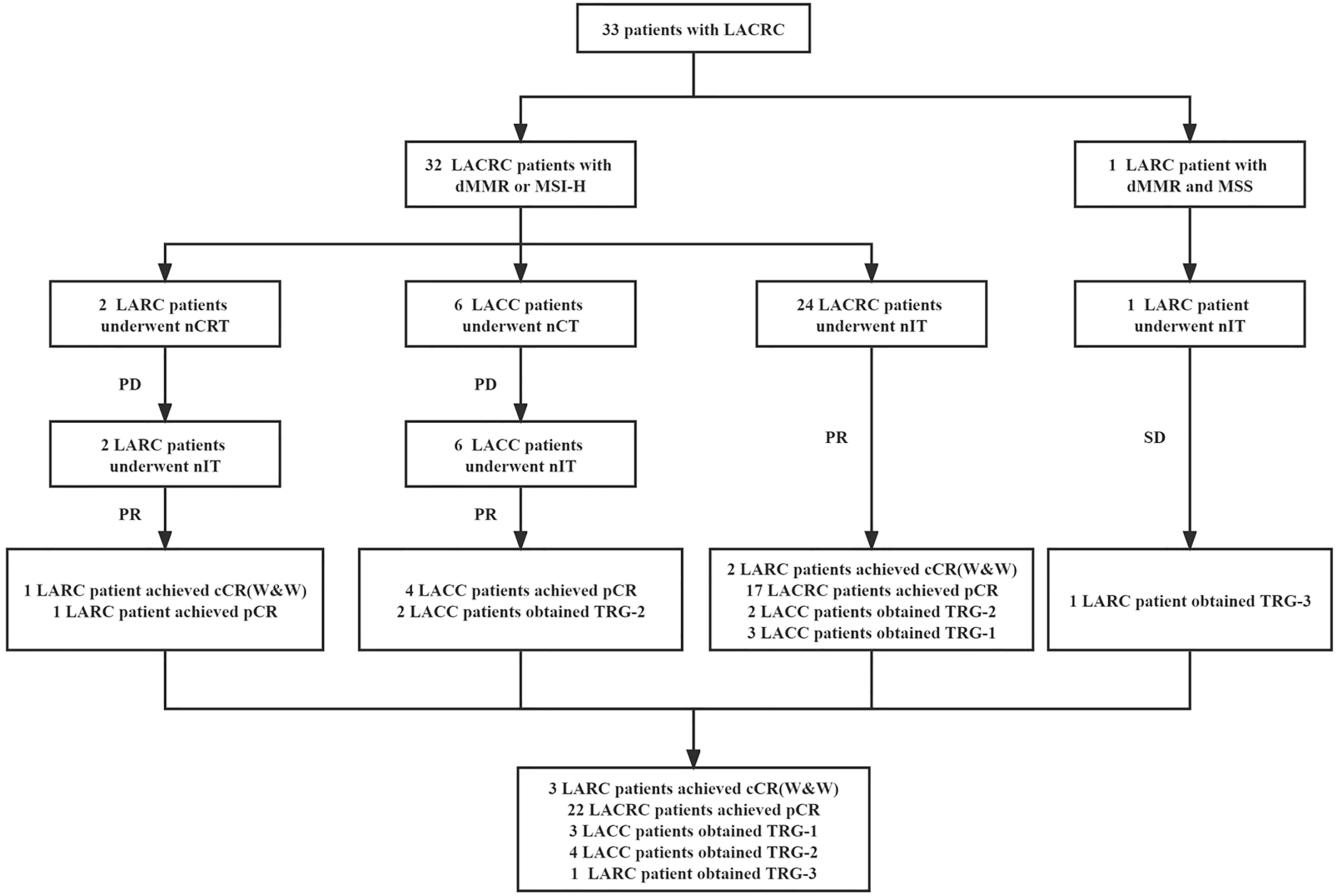
Study profile of nIT in patients with LACRC. LACRC, Locally advanced colorectal cancer; LARC, Locally advanced rectal cancer; LACC, Locally advanced colon cancer; dMMR, Mismatch repair-deficient; MSI-H, Microsatellite instability-high; MSS, Microsatellite stability; nIT, Neoadjuvant immunotherapy; nCRT, Neoadjuvant chemoradiotherapy; nCT, Neoadjuvant chemotherapy; cCR, Clinical complete response; pCR, Pathological complete response; W&W, Watch-and-wait; TRG, Tumor regression grade.

The study profile of nIT in patients with LACRC is shown in **Figure 1**. One patient with both dMMR and MSS LARC was excluded from efficacy evaluation. There were 17 males and 15 females, with a median age of 44 (23–62) years. Of all patients, 8 cases were rectal cancer and 24 cases were colon cancer (7 cases in ascending colon, 4 cases in hepatic flexure of colon, 4 cases in transverse colon, 2 cases in splenic flexure of colon, 2 cases in descending colon, 2 cases in sigmoid colon and 3 cases at the junction of rectum and sigmoid colon). 6 cases were clinical T3 stage and 26 cases were clinical T4 stage. 4 cases were clinical N0 stage and 28 cases were clinical N+ stage. There were 4 cases of clinical TNM in stage II and 28 cases in stage III. 9 cases were well differentiated, 13 cases were moderately differentiated and 10 cases were poorly differentiated. 15 patients were diagnosed with dMMR by IHC. 6 patients were detected as MSI-H by PCR. Another 11 cases were detected as both dMMR and MSI-H by IHC and PCR.

### Efficacy Evaluation of nIT With PD-1 Inhibitor

32 LACRC patients received neoadjuvant monoimmunotherapy with PD-1 blockade, of which 4 patients treated with pembrolizumab, 9 patients used sintilimab and 19 patients treated with tiselizumab. Among them, 2 LARC patients received nIT after the failure of nCRT and 6 LACC patients received nIT after the failure of nCT.

These 8 LACRC patients with second-line nIT were mainly because after the failure of first-line standard neoadjuvant therapies, our center detected their tumor tissues as dMMR by IHC or/and MSI-H by PCR, and developed a nIT regimen through multi-disciplinary team (MDT) discussion, which achieved significant ORR and pCR. Based on this, later, all newly diagnosed patients with CRC in our center were routinely tested for MMR or MSI status. If it is found to be dMMR or/and MSI-H, we began to try the exploration of first-line nIT. Therefore, the subsequent 24 LACRC patients were directly treated with first-line nIT when they were initially detected as dMMR/MSI-H.

**Figure 2:**
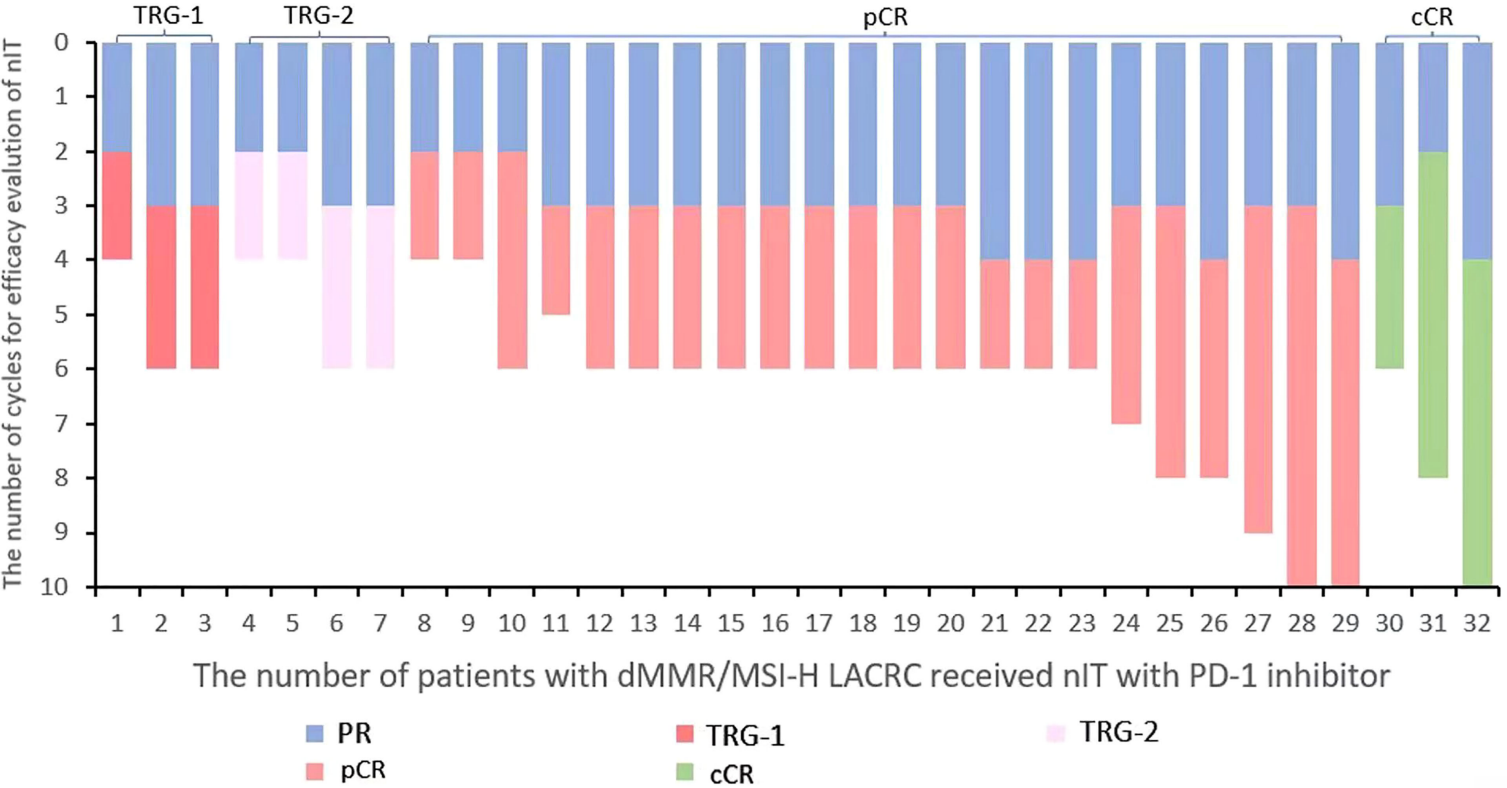
Waterfall plot of efficacy evaluation of nIT in patients with dMMR/MSI-H LACRC. LACRC, Locally advanced colorectal cancer; dMMR, Mismatch repair-deficient; MSI-H, Microsatellite instability-high; nIT, Neoadjuvant immunotherapy; PD-1, Programmed cell death protein⁃1; PR, Partial response; pCR, Pathological complete response; cCR, Clinical complete response.

The median number of cycles of 32 patients with dMMR/MSI-H LACRC receiving nIT with single-agent PD-1 inhibitor was 6 (4~10), that is, 18 weeks. The median number of cycles with efficacy evaluation reaching partial response (PR) was 3 (2~4) (**Figure 2**). Among 32 patients, three LARC patient achieved clinical complete response (cCR) and adopted the W&W strategy. The ORR of the remaining 29 LACRC patients with surgery was 100% (29/29), the pathological response rate was 100% (29/29), the MPR rate was 86.2% (25/29), and the pCR rate was 75.9% (22/29) (**Table 2**).

**Table 2:** Pathological outcomes of dMMR/MSI-H LACRC patients treated with nIT and surgery.

	nIT with PD-1 inhibitor group (n=29)	First-line nIT with PD-1 inhibitor group (n=22)	Second-line nIT with PD-1 inhibitor group (n=7)
ORR	29/29 (100% )	22/22 (100% )	7/7 (100% )
Pathological response rate	29/29 (100% )	22/22 (100% )	7/7 (100% )
MPR rate	25/29 (86.2%)	20/22 (90.9%)	5/7 (71.4%)
pCR rate	22/29 (75.9%)	17/22 (77.3%)	5/7 (71.4%)
TRG			
0	22/29 (75.9%)	17/22 (77.3%)	5/7 (71.4%)
1	3/29 (10.3%)	3/22 (13.6%)	0
2	4/29 (13.8%)	2/22 (9.1)	2/7 (28.6%)
3	0	0	0
Pathological T stage			
ypT0	22/29 (75.9%)	17/22 (77.3%)	5/7 (71.4%)
ypT1	3/29 (10.3%)	3/22 (13.6%)	0
ypT2	3/29 (10.3%)	2/22 (9.1%)	1/7 (14.3%)
ypT3	1/29 (3.5%)	0	1/7 (14.3%)
Pathological N stage			
ypN0	29/29 (100% )	22/22 (100% )	7/7 (100% )
ypN1	0	0	0
Pathological TNM stage			
ypT0N0M0	22/29 (75.9%)	17/22 (77.3%)	5/7 (71.4%)
ypT1N0M0-I	3/29 (10.3%)	3/22 (13.6%)	0
ypT2N0M0-I	3/29 (10.3%)	2/22 (9.1%)	1/7 (14.3%)
ypT3N0M0-IIA	1/29 (3.5%)	0	1/7 (14.3%)

LACRC, Locally advanced colorectal cancer; dMMR, Mismatch repair-deficient; MSI-H, Microsatellite instability-high; nIT, Neoadjuvant immunotherapy; ORR, Objective response rate; MPR, Major pathological response; pCR, Pathological complete response; TRG, Tumor regression grade; TNM, Tumor Node Metastasis.

In our study, one LARC patient who received nIT was both dMMR and MSS, so the patient was not included in the pathological evaluation. Three patients with dMMR/MSI-H low LARC achieved cCR after nIT and adopted the WW strategy, so these three patients were also excluded from pathological evaluation.

Further subgroup analysis demonstrated that 25% (8/32) of LACRC patients with dMMR/MSI-H received nCT or nCRT and then changed to nIT after ineffective.

Among them, one LARC patient achieved cCR and adopted the W&W strategy. The ORR of the other seven LACRC patients was 100% (7/7), the pathological response rate was 100% (7/7), the MPR rate was 71.4% (5/7), and the pCR rate was 71.4% (5/7); while the remaining 75% (24/32) of patients chose anti-PD-1 monotherapy as the initial first-line neoadjuvant therapy. Among them, two patients with LARC achieved cCR and adopted the W&W strategy. The ORR of the other 22 patients with LACRC was 100% (22/22), the pathological response rate was 100% (22/22), the MPR rate was 90.9% (20/22), and the pCR rate was 77.3% (17/22) (**Table 2**).

### IrAEs of nIT With PD-1 Inhibitor

The incidence of irAEs was 37.5% (12/32) during the nIT phase. Among them, the incidence of grade 1 irAEs was 31.25% (10/32), and grade 2 irAEs was 6.25% (2/32).

Meanwhile, after receiving nIT with single-agent PD-1 inhibitor, no grade 3 or above irAEs occurred in all patients. Furthermore, seven LACRC patients who achieved pCR after nIT and surgery did not receive postoperative adjuvant immunotherapy. While in the stage of adjuvant immunotherapy, the incidence of irAEs was 27.3% (6/22), all of which were grade 1 (**Table 3**).

**Table 3:** Immune-related adverse events (irAEs) and surgical-related adverse events (srAEs).

	nIT and surgery group (n=29)		nIT and W&W group (n=3)	
	Grade 1	Grade 2	Grade 1	Grade 2
	irAEs during the neoadjuvant phase			
Obstruction	1	1	0	0
Hyperthyroidism	1	0	0	0
Nausea	1	0	0	0
Fatigue	1	0	1	0
Aminotransferase increased	1	0	0	0
Abdominal pain	1	0	0	0
Pruritus or rash	0	1	0	0
Decreased appetite	1	0	0	0
Arthralgia or myalgia	1	0	0	0
Fever	0	0	1	0
Total	8/32 (25%)	2/32 (6.25%)	2/32(6.25%)	0
	**srAEs**			
Incision infection	0	1		
Intraoperative haemorrhage	1	0		
Postoperative haemorrhage	1	0		
Total	2/29 (6.9%)	1/29 (3.4%)		
**Surgery and adjuvant immunotherapy group (n=22)**				
**irAEs during the adjuvant phase**				
Dry mouth	1	0		
Dizziness	1	0		
Nausea	1	0		
Somnipathy	1	0		
Decreased appetite	1	0		
Pruritus or rash	1	0		
Total	6/22 (27.3%)	0		

nIT, Neoadjuvant immunotherapy; W&W, Watch-and-wait; irAEs, Immune-related adverse events; srAEs, Surgical-related adverse events.

one LARC patient who received nIT in this study was both dMMR and MSS, so the patient was not included in the baseline analysis. Seven patients with LACRC who achieved pCR after nIT and surgery did not receive postoperative adjuvant immunotherapy.

### Operation and srAEs After nIT With PD-1 Inhibitor

The median time from the last nIT to operation was 27 (16~42) days. All 29 patients underwent radical tumor resection. Among them, radical resection of rectal cancer was performed in 4 cases, radical resection of colon cancer in 24 cases, and resection of rectal cancer combined with other organs in 1 case. There were no delayed radical surgery due to irAEs in all patients. Intraoperative bleeding, postoperative bleeding and incision infection occurred in three patients (10.3%) respectively (**Table 3**). The other 26 patients had no srAEs such as intestinal obstruction, anastomotic bleeding, anastomotic stenosis and anastomotic fistula and so on.

### IrAEs of Adjuvant Immunotherapy With PD-1 Inhibitor

Among the 29 patients who underwent surgery, 22 patients received adjuvant immunotherapy, while 7 patients did not received. The incidence of irAEs in these 22 patients during adjuvant immunotherapy was 27.3% (6/22) (**Table 3**). Whether during nIT or adjuvant immunotherapy, all irAEs were grade 1~2, mainly gastrointestinal reactions and skin adverse reactions. No irAEs of grade 3 or above were found. All irAEs recovered after symptomatic treatment during immunotherapy.

### Follow Up Results

The last follow-up date was March 31, 2022. The median follow-up time of all 32 patients was 14 (3–28) months. No local recurrence or distant metastasis was found in all LACRC patients.

## Discussion

Patients with LACRC have a higher risk of recurrence and metastasis. Neoadjuvant therapies can improve the prognosis to a certain extent. Notwithstanding, the sensitivity of dMMR/MSI-H LACRC to nCT or nCRT remains low. The emergence of immunotherapy has brought hope to these patients. Immunotherapy was listed as the top ten scientific progress by *Science* in 2013. Based on the study of KEYNOTE-016 in 2015, it is confirmed that dMMR/MSI-H is a biomarker for the efficacy of immunotherapy, thus ushering in a novel era of immunotherapy in the field of CRC, and opening a new journey of precision immunotherapy in the era of precision medicine (18). Based on the results of CHECKMATE-142 and KEYNOTE-177 trials, the FDA has successively approved nivolumab ± ipilimumab or pembrolizumab for the second-line and first-line treatment of dMMR/MSI-H mCRC (19–22).

Whether immunotherapy can be used as neoadjuvant therapy for LACRC has become a hot topic. The NICHE study has demonstrated shocking results, marking the opening of the door of nIT for mCRC, opening up a novel treatment approach for patients with dMMR/MSI-H LACRC (10). Meanwhile, the lastest PICC study is a driving research on the application of nIT in dMMR/MSI⁃H LACRC (11). The NCCN guideline recommended universal screening for Lynch syndrome (LS) in CRC patients with dMMR/MSI-H (23). Subsequently, the NCCN guidelines (v1.2021) for colon cancer (24) and rectal cancer (25) recommend that all newly diagnosed CRC patients should be tested for MMR proteins expression (including MLH1, MSH2, MSH6 and PMS2) or MSI (including BAT25, BAT26, D5S346, D2S123 and D17S250) status. The guidelines also recommend nivolumab ± ipilimumab or pembrolizumab (preferred) as an option for preoperative neoadjuvant treatment of resectable dMMR/MSI⁃H mCRC (24, 25). This is the first time the NCCN recommended an immunotherapy as a neoadjuvant therapy for CRC. In the newest NCCN guideline (v1.2022) for colon cancer (26) just released this year, an updated point is that the neoadjuvant therapy regimen of nivolumab ± ipilimumab or pembrolizumab is considered as an option for patients with dMMR/MSI⁃H cT4b colon cancer.

Nevertheless, the safety and effectiveness of immunotherapy in the neoadjuvant setting for dMMR/MSI-H LACRC still need more clinical exploration and verification.

In our study, the median number of cycles of 32 patients receiving nIT with PD-1 inhibitor was 6 (4 ~ 10), and the median number of cycles with efficacy evaluation reaching partial response (PR) was 3 (2 ~ 4). One LARC patient with both dMMR and MSS was excluded from efficacy evaluation. Among 32 patients with dMMR/MSI-H LACRC, three LARC patients achieved cCR and adopted the W&W strategy. The ORR of the other 29 LACRC patients was 100% (29/29), the pathological response rate was 100% (29/29), the MPR rate was 86.2% (25/29), and the pCR rate was 75.9% (22/29). Subgroup analysis demonstrated that the pCR rate of 22 patients with first-line nIT was 77.3% (17/22), while that of 7 patients with second-line nIT was 71.4% (5/7). No significant difference in pCR rate was found between the two nIT groups. It can be seen that nIT could achieved high rate of tumor down-staging and pCR for patients with dMMR/MSI-H LACRC, regardless of whether they have received nCT or nCRT before.

The main reason is that CRC patients with middle- and early-stage have relatively sound immune systems, and their tumor burdens are generally not as severe. These LACRC patients express many tumor neoantigens, and this can increase the activity of anti-tumor immune T cells, followed by dispersal throughout the body and removal of micro-metastases (27). After a multi-line treatment, the immune microenvironment and physical state of LACRC patients has varying degrees of dysfunction. Therefore, a better response could theoretically be obtained through the earlier application of immunotherapy.

Further comparative analysis indicated that 20 patients with dMMR stage I ~ III colon cancer included in NICHE study were treated with nivolumab [3 mg/kg (day1, day15)] combined with ipilimumab [1 mg/kg (day1)] for 4 weeks, and the pCR rate was 60.0% (12/20) (10), and 17 patients with dMMR/MSI⁃H LACRC included in PICC study received single-agent nIT with treprizumab (200mg, q3w) for 6 cycles (18 weeks), and the pCR rate was 64.7% (11/17) (11). We found that the pCR rate (75.9%) of our study was significantly higher than the reported data of the above two nIT trials (10, 11). Among the 29 patients treated with surgery in our study, the median number of preoperative treatment cycles of single-agent nIT with PD-1 inhibitor was also 6 (18 weeks), and the median time from the last nIT to operation was 27 (16 ~ 42) days. Thus, the high pCR rate in our study may be related to more cycles of preoperative immunotherapy, longer treatment intervals, and the tailing effect of immunotherapy. In addition, the patients selection may also be related.

With regard to adjuvant therapy in this study, of the 29 patients who underwent surgery, 22 with clinical stage III received only adjuvant immunotherapy without combined chemotherapy and the remaining 7 including 4 cases of clinical stage II and 3 cases of stage III did not received any adjuvant therapy including adjuvant immunotherapy or chemotherapy. The postoperative pathological results of these 7 patients achieved pCR. Among them, 4 patients were stage II, of which 1 received 6 cycles, 1 received 7 cycles and 2 received 8 cycles of nIT, and the remaining 3 patients were stage III, of which 1 received 9 cycles and 2 received 10 cycles of nIT. It can be seen that these 7 patients received a long cycle of nIT and achieved pCR, which are the main reason why they did not receive postoperative immunotherapy or chemotherapy. Among the 22 patients with clinical stage III, 4 patients obtained TRG-2 in pathological results, of which 2 received 4 cycles, and 2 received 6 cycles of nIT, 3 patients obtained TRG-1, of which 1 received 4 cycles, 2 received 6 cycles of nIT, and the remaining 15 patients achieved pCR, of which 2 received 4 cycles, 1 received 5 cycles and 12 received 6 cycles of nIT. It can be found that the cycle of these 22 patients receiving nIT is relatively short, and about 32% of the patients did not reach pCR, which are the main reason for them to receive adjuvant therapy.

Patients with dMMR/MSI⁃H pathological stage II CRC have good prognoses in general. The current guideline recommendations is that adjuvant chemotherapy is not needed for patients with low-risk stage II CRC. Meanwhile, patients with dMMR/MSI⁃H pathological stage III CRC who received adjuvant chemotherapy have better prognoses than MSS patients (28, 29). However, only adjuvant immunotherapy was chose for postoperative treatment regime in those 22 patients with clinical stage III, and no or not combined chemotherapy was chose. The reason is that preoperative nIT has achieved significant tumor regression and less irAEs. Actually, the principle of “effective drugs without changing drugs” is followed. Can adjuvant immunotherapy further improve the prognosis of dMMR/MSI-H LACRC patients? Several ongoing phase III prospective clinical trials are trying to answer this question. Among them, the ATOMIC study (NCT02912559) is a clinical study of atezilizumab combined with chemotherapy versus chemotherapy in the adjuvant treatment of dMMR stage III CRC, and the primary endpoint is disease-free survival(DFS). The POLEM trial (NCT03827044) is a multicenter phase III randomized clinical trials(RCTs) of avelumab combined with fluoropyrimidine in the adjuvant treatment of dMMR or POLE nucleic acid exonuclease mutations. Another Phase III clinical trial (NCT⁃03803553) will evaluate the efficacy of nivolumab in patients with MSI⁃H CRC after standard adjuvant chemotherapy. We look forward to the outcomes of the above and more postoperative adjuvant immunotherapy studies.

Meanwhile, three patients with dMMR/MSI-H low LARC achieved cCR after nIT and adopted the W&W strategy in our study. The last time they received immunotherapy was 6 months, 9 months and 12 months respectively, and no local recurrence or distant metastasis was found during follow-up. The breakthrough efficacy of nIT for many patients with dMMR/MSI-H LACRC provides colorectal oncologists with great hope, especially for patients with dMMR/MSI-H low LARC. The published studies manifested that nIT was associated with less risk of anal sphincter dysfunction, sexual dysfunction, abnormal fecal control, and bladder dysfunction than traditional nCT and nCRT (30, 31). From the extremely high pCR rate obtained by nIT and the characteristics of lasting benefits once immunotherapy is effective, it can be inferred that patients with dMMR/MSI-H low LARC who achieve cCR after nIT are ideal people to adopt the W&W strategy, but it is undeniable that long-term follow-up and high-level evidences are needed to support this viewpoint.

Additionally, the LACRC patients in our study were either determined by IHC detection of MMR proteins expression status or by PCR detection of MSI status. Remarkably, one LACC patient was identified as dMMR (PMS2 protein was missing) by IHC but confirmed to be MSS (five loci were not changed) by PCR testing, that is, the patient’s MSI and MMR status were inconsistent. The reasons for the simultaneous detection of dMMR and MSS may be that the loss of some MMR protein is compensated by their function, or the tumor heterogeneity caused by the methylation of MLH1 promoter, which will affects the judgment of results (32). Although this patient obtained the opportunity of radical resection after 6 cycles of neoadjuvant anti-PD-1 monotherapy, both the preoperative imaging and postoperative pathological results suggested poor tumor regression, and the patient was the only case with TRG of grade 3 in this study.Therefore, the efficacy of immunotherapy may be reduced when the MMR protein expression and MSI status are inconsistent. Studies have demonstrated that poor response to ICIs in several patients with dMMR may be related to mis-judgment of their MMR status (19, 33). Although the study have pointed out high sensitivity and specificity for MMR protein expression status detected by IHC and MSI status identified by PCR or next-generation sequencing (NGS), and the consistency of the three test results > 95% (34), we believe that for patients with dMMR/MSI⁃H LACRC who are recommended for nIT, it is the most consistent with the principle of accurate diagnosis to determine both the status of MMR protein expression and MSI before receiving nIT. Interestingly, several clues can be found from our study and previous study (35), such as young patients (< 40 years old), huge tumor volume, typical lynch syndrome, including family history of CRC or endometrial cancer, which can indirectly assist in suggesting the high possibility of dMMR/MSI⁃H.

The incidence of irAEs in 32 patients during nIT was 37.5% (12/32), while the incidence of irAEs in 22 patients during postoperative adjuvant immunotherapy was 27.3% (6/22), all of which were grade 1~2, mainly gastrointestinal reactions and skin adverse reactions. No patient had grade 3 or above irAEs, which proved that the nIT regimen with single-agent PD-1 blockade was relatively safe in general. Nonetheless, larger-sample prospective studies are warranted. Moreover, all irAEs in our study have been reported in other immunotherapy studies (10, 11, 35). In this study, 29 patients underwent radical surgery and achieved R0 resection. There was no delay in radical surgery due to irAEs in all cases. Only 10.3% (3/29) of the srAEs occurred, and recovered after symptomatic treatment. At last, no perioperative death and no local recurrence or distant metastasis was found in all patients by now. Consequently, we can deem that the early application of immunotherapy does not increase the additional risk of surgery.

In addition, our research team found a phenomenon, that is, when re-examinating the colonoscopy, it was found that the tumor regression of many patients was very significant, and even the tumor could not be seen by the naked eye. Meanwhile, compared with the colonoscopy before treatment, the intestinal cavity at the tumor here was so narrow that the mirror body was often unable to pass through. Furthermore, the serous surface contracture at the tumor of the postoperative specimen can be seen. When the intestinal tube is opened, the intestinal cavity contracture at the tumor is narrow, and the mucosal surface tissue is hard, but the submucosa and muscular tissue are thickened, the texture is soft, and a large amount of mucus or necrotic components can be seen. The reason for the change may be that the histological changes of intestinal wall after ICIs treatment include thickening of lamina propria, shortening of villi, infiltration of neutrophils and apoptosis of crypt glands in the epithelial layer, and there are few lymphocytes in the epithelial layer (36). Of course, these findings are anecdotes or subjective feelings, which are unlikely to be quantified as objective indicators at present.

Our study is a rare, relatively large-sample single-center, real-world study of neoadjuvant monoimmunotherapy in dMMR/MSI-H LACRC, covering initially unresectable or resectable stage II-III colon cancer or rectal cancer. Despite diverse treatment methods, such as various types of PD-1 inhibitors, and varying cycles of nIT, and a few patients received nCT or nCRT before nIT, all patients received single-agent anti-PD-1 antibody as nIT strategy, and achieved ideal tumor regression with low irAEs and srAEs. Of course, this study also has several limitations. First, there is a lack of long-term follow-up data on quality of life and survival. Second, this study is a single-center, retrospective, so the level of evidence-based medicine is not high enough. Third, the tumor characteristics of patients in this study were inconsistent with those reported in other large-sample study (37). For example, the proportion of BRAF mutations in this study was relatively low, while the proportion of isolated MSH2 loss was relatively high, about 20%. There were only 17 patients detected by PCR and 26 patients detected by IHC. Hence, there may be relatively few cases in our study, resulting in a certain degree of deviation in the proportion. Hence, we will continue to expand the sample size in order to make more convincing comparisons and draw more rigorous conclusions.

## Conclusions

In conclusion, the nIT regimen of single-agent PD-1 inhibitor is sufficiently secure and remarkably effective for patients with dMMR/MSI-H LACRC. Large-scale, multi-center prospective RCTs are warranted to further verify the long-term efficacy and safety, optimal number of cycles and predictive biomarkers of nIT for LACRC. Meanwhile, it can be predicted that the W&W strategy is a promising treatment approach for managing patients with dMMR/MSI-H low LARC who achieve cCR after nIT and will help promote colorectal surgery into a new individualized and precise “non-invasive” era.

## Data Availability Statement

The raw data supporting the conclusions of this article will be made available by the authors, without undue reservation.

## Ethics Statement

The studies involving human participants were reviewed and approved by The ethics committee of Yunnan cancer hospital/The Third Affiliated Hospital of Kunming Medical University, China. The patients/participants provided their written informed consent to participate in this study.

## Author Contributions

All authors contributed to the study conception and design. Drafting the work and/or revising it critically: XZ, RY, and TW. Final approval of the version to be published: XZ, and YuL. All authors contributed to the article and approved the submitted version.

## Funding

This study was supported by the National Natural Science Foundation of China (82060542 to CD), and the Yunnan Provincial Department of Education Science Research Fund Project (2022J0227 to XZ).

## Conflict of Interest

The authors declare that the research was conducted in the absence of any commercial or financial relationships that could be construed as a potential conflict of interest.

## Publisher’s Note

All claims expressed in this article are solely those of the authors and do not necessarily represent those of their affiliated organizations, or those of the publisher, the editors and the reviewers. Any product that may be evaluated in this article, or claim that may be made by its manufacturer, is not guaranteed or endorsed by the publisher.
